# Break up the band: Laparoscopic Adjustable Gastric Banding-associated Discitis and Osteomyelitis

**DOI:** 10.5811/cpcem.2019.11.44879

**Published:** 2020-01-21

**Authors:** Scott Meester, Christopher Hogrefe

**Affiliations:** University of Iowa Hospitals and Clinics, Department of Emergency Medicine, Iowa City, Iowa

## Abstract

Obesity is an epidemic that adversely affects millions of Americans. In 2017, the Center for Disease Control and Prevention reported that 93.3 million Americans suffer from obesity.^1^ Many individuals have undergone laparoscopic adjustable gastric banding (LAGB) procedures in order to lose weight. The procedure is thought to be safe with complication rates reported as low as 1.6% following surgery.^2^ We present a case of LAGB-associated discitis and osteomyelitis 20 years after placement and examine the current literature on the complication rates of bariatric surgery along with the rare injuries following LAGB placement.

## INTRODUCTION

Morbid obesity is an epidemic affecting 93.3 million individuals in the United States, or almost 40% of the population.^1^ Multiple medical and surgical treatment options exist to combat obesity. One such surgical option is the laparoscopic adjustable gastric banding (LAGB) procedure. In 2017 alone, 228,000 individuals underwent bariatric surgery with 2.77% involving an LAGB procedure.^2^ Complications have been reported with this surgery, including infection, bleeding, and erosion of the gastric band through gastric wall. We report a complication that has not been previously detailed in the literature, specifically an LAGB catheter eroding into the spine resulting in discitis and osteomyelitis.

## CASE REPORT

The patient is a 55-year-old gentleman with a past medical history remarkable for hypertension, methamphetamine abuse, epidural and paraspinal abscesses in 2018, and an LAGB procedure in 1998 that presented to the Emergency Department with back pain. The patient reported a two month history of back pain that was progressively worsening. He described sharp lower midline back pain with right lower extremity weakness. The patient’s initial vital signs revealed a temperature of 36.6°C, heart rate of 62 beats per minute, a blood pressure of 135/82 millimeters of Mercury (mmHg), and an oxygen saturation of 99% on room air. Skin exam was notable for a well-healed 6 centimeters surgical wound to the right side of the lumbar spine. The patient had full range of motion of his upper and lower extremities. Sensation was intact in the bilateral upper and lower extremities. Strength in his right hip and right knee were 3/5, while strength in the left lower extremity was 5/5. Straight leg raise testing was positive bilaterally, with the right side being more painful than left. At that juncture the working differential diagnosis included epidural abscess, epidural hematoma, lumbar radiculopathy, and/or worsening osteomyelitis. Laboratory studies were significant for hemoglobin of 8.6 gram/deciliter (g/dL) [11.9 – 15.5 g/dL], a white blood cell count of 9.0 thousand/millimeter^3^ (K/mm^3^) [3.7–10.5 K/mm^3^], an erythrocyte sedimentation rate of 122 millimeter/hour (mm/hr) [0–20 mm/hr], and a C-reactive protein of 4.4 milligram/deciliter (mg/dL) [ ≥ 0.5 mg/dL].

A computed tomography (CT) scan of the abdomen and pelvis was performed given the patient’s persistent pain and recent spinal epidural abscess drainage at an outside hospital. CT scan showed concerns for continued abscesses. Given abnormal neurological exam and abscesses, a magnetic resonance imaging (MRI) of the thoracic and lumbar spine was secured (Image). This revealed an LAGB ring component eroding through the gastric wall with localized pneumatosis. The catheter from the LAGB appeared to have passed through the retroperitoneum and the left psoas into the central spinal canal through the second lumbar (L2)–L3 vertebral body resulting in discitis and osteomyelitis with surrounding abscesses. The transferring hospital treated the patient with a 30 milligram/kilogram (mg/kg) fluid bolus and started intravenous metronidazole, vancomycin, and fluconazole. The patient’s pain was treated with intravenous dilaudid. He was subsequently transferred for an orthopaedic spine evaluation, as this was not available locally.

Given the aforementioned imaging, the diagnosis of L2–L3 discitis and osteomyelitis was made along with both spinal canal and iliopsoas foreign bodies consisting of hardware from the LAGB. The patient was admitted to the surgical intensive care unit, after which he underwent laparoscopic and endoscopic removal of the gastric LAGB and spinal fusion of the twelfth thoracic vertebra (T12) to L5 with decompression of the spine. His hospital course was complicated by septicemia treated with intravenous ertapenem and fluconazole. The patient remained in the hospital nineteen days and was discharged home with resolved lower extremity weakness and overall normal neurological status. The patient did, however, continue to experience gradually improving lower back pain following his surgery, which was treated with oral medications.

## DISCUSSION

Gastric bypass surgery is a popular procedure for the morbidly obese population to help promote weight loss. The American Society for Metabolic and Bariatric Surgery estimates that approximately 228,000 individuals underwent bariatric surgery in 2017. Of these individuals, 2.77% underwent LAGB placement.^3^ Multiple studies have examined the revision rates of LAGB and complications from LAGB placement. A study looking at approximately 32,000 perioperative bariatric surgery patients examined the morbidity and complications following this operation. In LAGB procedures specifically, they found the total complication rate to be 1.6%, which included infection, hemorrhage, and/or cardiovascular complications. Bleeding during the procedure was the most common complication.^2^ However, multiple studies following patients over time revealed a complication rate of 15.0% – 34.17%. 4,5 One study in particular found an explant rate secondary to complication of 8.74%. 6 Most complications were not statistically significant; they included abscess, atelectasis, depression, internal complications, port leak or band removal, displacement, slippage, and numerous other complications.^4^ However, no examined reviews of LAGB complications reported discitis and/or osteomyelitis of the spine.

**CPC-EM Capsule**

What do we already know about this clinical entity?

Laparoscopic adjustable gastric banding (LAGB) comes with complications such as infection, bleeding, and erosion of the gastric band through the gastric wall.

What makes this presentation of disease reportable?

LAGB-associated discitis and osteomyelitis is a complication of LAGB placement that has yet to be reported in literature.

What is the major learning point?

LAGB patients with back or abdominal pain may be suffering from further pathology such as discitis or osteomyelitis secondary to hardware migration.

How might this improve emergency medicine practice?

Providers seeing patients with persistent and significant back or abdominal pain should consider further imaging to rule out rare pathology.

One of the most commonly mentioned and reviewed complications of the LAGB procedure is erosion. Niville et al. followed 301 patients for two years after an LAGB procedure. A total of 5 patients (1.66%) developed erosions into the stomach wall and required LAGB removal.^7^ This suggests that migration of the LAGB should be considered in anyone presenting with abdominal pain following an LAGB procedure. Although no cases of osteomyelitis of the spine have been reported previously, pericardial effusion after a postoperative infection of an LAGB has been reported in one case.^8^

Based on these studies, it appears that although complication rates of LAGB procedures can be as high as 34%, erosion and migration of LAGB are less commonly seen. However, if a patient presents with back pain in the context of a previous LAGB placement, a rare complication that must be considered is osteomyelitis secondary to migration of LAGB catheter into the spinal space.

## CONCLUSION

Laparoscopic adjustable gastric banding-associated spinal discitis and osteomyelitis are rare complications of LAGB erosion. Symptoms such as back pain, radicular pain, numbness, or tingling, lower extremity motor weakness or loss of reflexes, and disturbed bowel or bladder function should be evaluated further with imaging. An MRI with contrast of the spine should be considered in the setting of neurological exam findings and continued symptoms despite adequate treatment in order to better characterize the spine. Although this is the first reported such case, it is worth remembering that patients with significant back pain and neurological findings warrant further investigation into the etiology of their symptoms.

## Figures and Tables

**Image:**
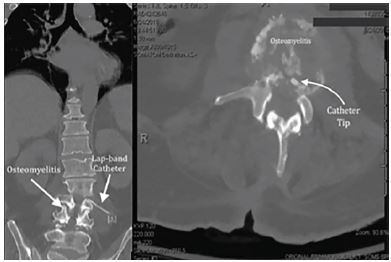
Computed tomography of the lumbar spine, (left) coronal view and (right) axial view demonstrating erosion of the laparoscopic gastric banding catheter into the spinal area and associated osteomyelitis.
